# Circular RNA Profiling and Bioinformatic Modeling Identify Its Regulatory Role in Hepatic Steatosis

**DOI:** 10.1155/2017/5936171

**Published:** 2017-06-21

**Authors:** Xing-Ya Guo, Chong-Xin He, Yu-Qin Wang, Chao Sun, Guang-Ming Li, Qing Su, Qin Pan, Jian-Gao Fan

**Affiliations:** ^1^Department of Gastroenterology, Xinhua Hospital, Shanghai Jiaotong University School of Medicine, Shanghai 200092, China; ^2^Department of Endocrinology, Xinhua Hospital, Shanghai Jiaotong University School of Medicine, Shanghai 200092, China; ^3^Shanghai Key Laboratory of Children's Digestion and Nutrition, Shanghai 200092, China

## Abstract

Circular RNAs (circRNAs) exhibit a wide range of physiological and pathological activities. To uncover their role in hepatic steatosis, we investigated the expression profile of circRNAs in HepG2-based hepatic steatosis induced by high-fat stimulation. Differentially expressed circRNAs were subjected to validation using QPCR and functional analyses using principal component analysis, hierarchical clustering, target prediction, gene ontology (GO), and pathway annotation, respectively. Bioinformatic integration established the circRNA-miRNA-mRNA regulatory network so as to identify the mechanisms underlying circRNAs' metabolic effect. Here we reported that hepatic steatosis was associated with a total of 357 circRNAs. Enrichment of transcription-related GOs, especially GO: 0006355, GO: 004589, GO: 0045944, GO: 0045892, and GO: 0000122, demonstrated their specific actions in transcriptional regulation. Lipin 1 (LPIN1) was recognized to mediate the transcriptional regulatory effect of circRNAs on metabolic pathways. circRNA-miRNA-mRNA network further identified the signaling cascade of circRNA_021412/miR-1972/LPIN1, which was characterized by decreased level of circRNA_021412 and miR-1972-based inhibition of LPIN1. LPIN1-induced downregulation of long chain acyl-CoA synthetases (ACSLs) expression finally resulted in the hepatosteatosis. These findings identify circRNAs to be important regulators of hepatic steatosis. Transcription-dependent modulation of metabolic pathways may underlie their effects, partially by the circRNA_021412/miR-1972/LPIN1 signaling.

## 1. Introduction

Hepatic steatosis reflects a pathological disorder characterized by excess triglyceride (TG) accumulation (≥5% of volume or weight) in the liver and demonstrates close association with obesity, type 2 diabetes, hyperlipidemia, and other components of metabolic syndrome with limited exceptions (i.e., alcohol abuse, chronic hepatitis C) [1–3]. Serving as the hallmark of nonalcoholic fatty liver disease (NAFLD), hepatic steatosis predisposes patients to nonalcoholic steatohepatitis (NASH), liver fibrosis/cirrhosis, and the final outcome of hepatocellular carcinoma (HCC) [4]. High incidence of extrahepatic death and disability (i.e., cardiovascular events, cerebral apoplexy, and cancers) also takes place in the population with hepatic steatosis [5–7]. Recent decades have witnessed a dramatic prevalence of hepatic steatosis increase worldwide, ranging in 24%–46% in the western countries and 7.9%–54% in the Asia-Pacific area [8–11], which suggests the coming emergency and serious burden of public health. For the sake of our limited understanding in its mechanisms, hepatic steatosis is still beyond the reach, to a large extent, of clinical interference.

Circular RNA (circRNA), a class of noncoding RNAs with the linking of 3′ and 5′ ends, used to be regarded as the nonfunctional byproducts of mRNA splicing [12]. However, characteristics of circRNA, including tissue- and development-specific expression, enrichment of miRNA response element (MRE), and resistance to both RNase R and RNA exonuclease, highlight its potential role in the gene regulation of eukaryotes [13]. Studies indeed prove that circ-HIPK3 governs the proliferation of seven kinds of cancer cells via binding to miR-124 [14], while formation of circ-Foxo3-p21-CDK2 ternary complex induces the cell cycle arrest in noncancer cells [15]. Cdr1as and mmu-circRNA-015947 are well described to act as the regulator in insulin secretion and cerebral ischemia-reperfusion injury, respectively [16, 17]. Moreover, HRCR protects the heart from pathological hypertrophy and heart failure by targeting miR-223 [18]. Thus circRNAs are of physiological and pathological importance in various species, mainly on the basis of being complimentary to the “seed sequence” of miRNAs. But the effect of circRNAs on hepatic steatosis remains to be clarified until now.

We, therefore, investigated the expression profile of circRNAs in HepG2-based hepatic steatosis induced by high-fat stimulation. Targetome of these differentially expressed circRNAs was established at both miRNA and mRNA levels via complementation-dependent target prediction. Then gene ontology (GO) terms and pathway annotation were employed for functional analysis on the basis of circRNAs' targetome. Integrating significant GOs and the underlying pathways, circRNA-miRNA-mRNA network was constructed to identify the key circRNAs, and finally their action model, in steatogenesis.

## 2. Materials and Methods

### 2.1. Establishment of Hepatic Steatosis by High-Fat Stimulation

HepG2 cells (Cell Bank of Type Culture Collection, China), the cell line of human hepatocarcinoma, in the exponential phase were randomized into groups of control (*n* = 3) and model (*n* = 3), respectively, and seeded in the 96-well plate at 4 × 10^3^/well. In contrast to the control group cultured by Dulbecco's modified Eagle's medium (DMEM, HyClone Laboratories, Inc., USA), penicillin-streptomycin, and 10% fetal bovine serum (FBS, Gibco BRL, USA), HepG2 cell in the model group were incubated additionally with 0.5 mM free fatty acid (FFA, oleate : palmitate = 2 : 1; Sigma-Aldrich, USA) dissolved in dimethyl sulfoxide (DMSO) [19].

### 2.2. Oil Red-O Staining and Triglyceride Assay

After the treatment of FFA for 24 hours, both control and model groups were evaluated by Oil Red-O staining. In this assay, formaldehyde-fixed HepG2 cells were administrated using 0.5% Oil Red-O in isopropyl alcohol for 20 minutes and then counterstained using hematoxylin for 1 minute. TG level was also enzymatically measured in the HepG2 cells of both groups using TG assay kit (Applygen Technologies Inc., China) according to the manufacturer's instructions.

### 2.3. Microarray Hybridization for circRNA Profile

Total RNA, derived from both control and model groups, was extracted by the TRIzol reagent. The RNA purity and integrity of each sample were checked using light absorbance (260 nm/280 nm) and agarose gel electrophoresis, respectively. To remove linear RNAs and enrich circRNA, total RNA from each sample was treated with Rnase R. circRNA was then transcribed into fluorescent cRNA by random primer according to the Arraystar Super RNA Labeling protocol (Arraystar, Inc., USA) and hybridized onto the Arraystar Human circRNA Arrays V2.0 (Arraystar, Inc., USA) at 65°C for 17 hours. After washing, the arrays were subjected to scanning by Agilent Scanner G2505C (Agilent, USA) and data extraction by Agilent Feature Extraction software (Agilent, USA). Finally, differentially expressed circRNAs between two groups were filtered using fold change cutoff (>2, or <0.5) and statistical significance. Both unsupervised hierarchical clustering (Cluster 3.0) and TreeView analysis (Stanford University, USA) were performed to these differentially expressed circRNAs.

### 2.4. Quantitative Real-Time Polymerase Chain Reaction

Six differentially expressed circRNAs were selected at random and verified by Mir-X miRNA First Strand Synthesis Kit (Takara, Da Lian, China) using Applied Biosystems 7500 Real-Time PCR Detection Systems (Bio-Rad, USA). The circRNA levels, being normalized against GAPDH [20, 21], were evaluated by the 2^−ΔΔCt^ method [22]. Triplicates were performed for each sample in three independent experiments. Primers used for stem–loop RT-PCR are shown in Table 1.

### 2.5. Principal Component Analysis

Principal component analysis (PCA) was employed to investigate the overall relationships between circRNA expression and groups through dimensionality reduction and feature extraction. The analysis was conducted using R software 3.3.1 (R Development Core Team) [23–25].

### 2.6. GO Terms and Signal Pathway Annotation

Target miRNAs of the differentially expressed circRNAs were predicted using Arraystar's home-made miRNA target prediction software according to method of MRE-based circRNA/microRNA complementation (Arraystar Inc., USA) [26, 27]. mRNAs downstream to these miRNAs were pooled to construct the targetome by mirdb5.0 and targetscan7.1 databases [28]. Then, GO term analysis was applied to differentially expressed circRNAs on the basis of targetome using DAVID gene annotation tool (http://david.abcc.ncifcrf.gov/) [29]. In detail, two-side Fisher's exact test was used to classify the GO category, while the false discovery rate (FDR) was calculated to correct the *P* value [30]. Those GOs with *P* value < 0.01 and FDR < 0.01 were filtered to be of statistical significance.

Similarly, pathway analysis uncovered the significant pathways related to differential expressed circRNAs according to the annotation of Kyoto Encyclopedia of Genes and Genomes (KEGG) database (http://www.genome.jp/kegg/) [31]. The threshold of significance was defined by *P* value (<0.01) and FDR (<0.01) as mentioned above. The gene interaction within GOs and pathways, respectively, was investigated by databases of Ingenuity® Pathway Analysis (IPA), KEGG, and PubMed [32].

### 2.7. Bioinformatics Modeling for the Regulatory Network of circRNAs

Both the circRNA-miRNA and miRNA-mRNA interactions, as previously described by Targetscan and miRanda algorithm [28], were integrated to delineate the regulatory network of steatosis-related circRNAs. First, the adjacency matrix of circRNA and miRNA was given by

(1) $$\begin{array}{c} A=\left[\;a_{i,j}\;\right]. \end{array}$$

*a*_*i*,*j*_ represents the weight of relation between miRNA (*i*) and circRNA (*j*) [33]. Similar matrix of mRNA and miRNA was also obtained by their weighted relation. Second, the circRNA-miRNA-mRNA network was established on the basis of both adjacency matrixes by Cytoscape 3.1.0 software [34]. Additionally, mRNA sets of the significant GOs and pathways were cross-intersected so as to identify the critical genes that mediate circRNAs' pathway-dependent effects on biological processes. By the circRNA-miRNA-mRNA network, we uncovered the signaling cascade of circRNAs, miRNAs, and critical mRNAs with great importance to circRNAs' regulatory role in hepatic steatosis.

### 2.8. Statistics

All the results were expressed as mean ± standard deviation (SD). Statistical analysis was performed by Student's* t*-test for the comparison of different groups using GraphPad Prism 5.0 (GraphPad Software, La Jolla, CA) [35], whereas Fisher's exact test was employed to filter the significant GOs and pathways using R software 3.3.1 (R Development Core Team). In both cases,* P* value of less than 0.05 (two-tailed) was considered statistically significant.

## 3. Results

### 3.1. Dysregulation of circRNA Profile in HepG2-Based Hepatic Steatosis

**Figure 1:**
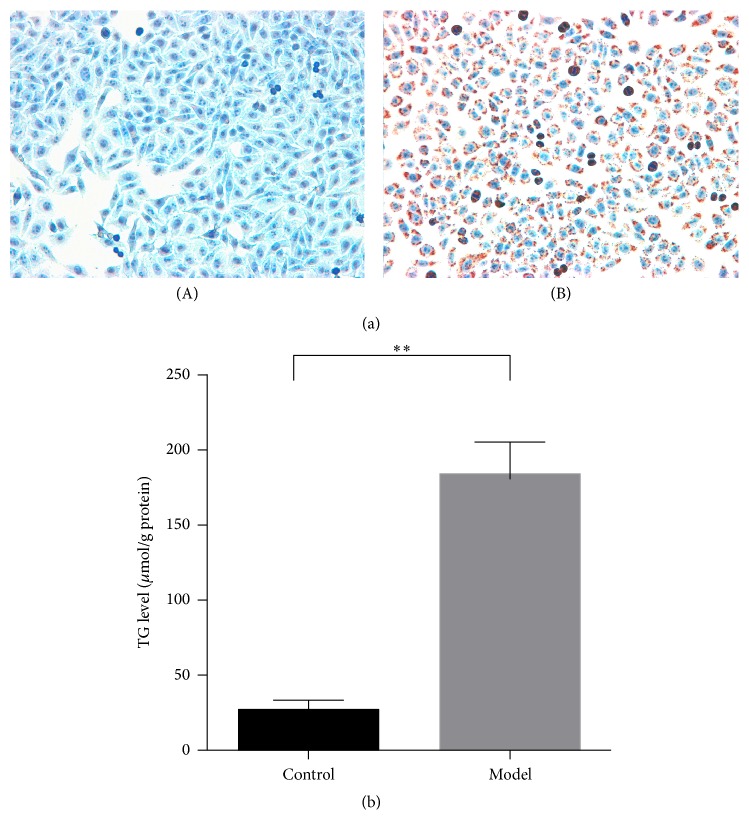
Establishment of hepatic steatosis in HepG2 cells by high-fat stimulation. (a) Oil Red-O staining for the HepG2 cells in normal (A) and model (B) groups (200x). (b) Triglyceride assay for the normal and model groups. The presented results are expressed as means ± SD. ^*∗∗*^*P* < 0.01.

When compared to those of the normal group, HepG2 cells of the model group demonstrated plentiful lipid droplets positive to Oil Red-O staining after the high-fat stimulation for 24 hours (Figure 1(a)). In consistence to the phenotype alternation, significant upregulation in TG content characterized the model group (Figure 1(b)), reflecting the establishment of hepatic steatosis.

**Figure 2:**
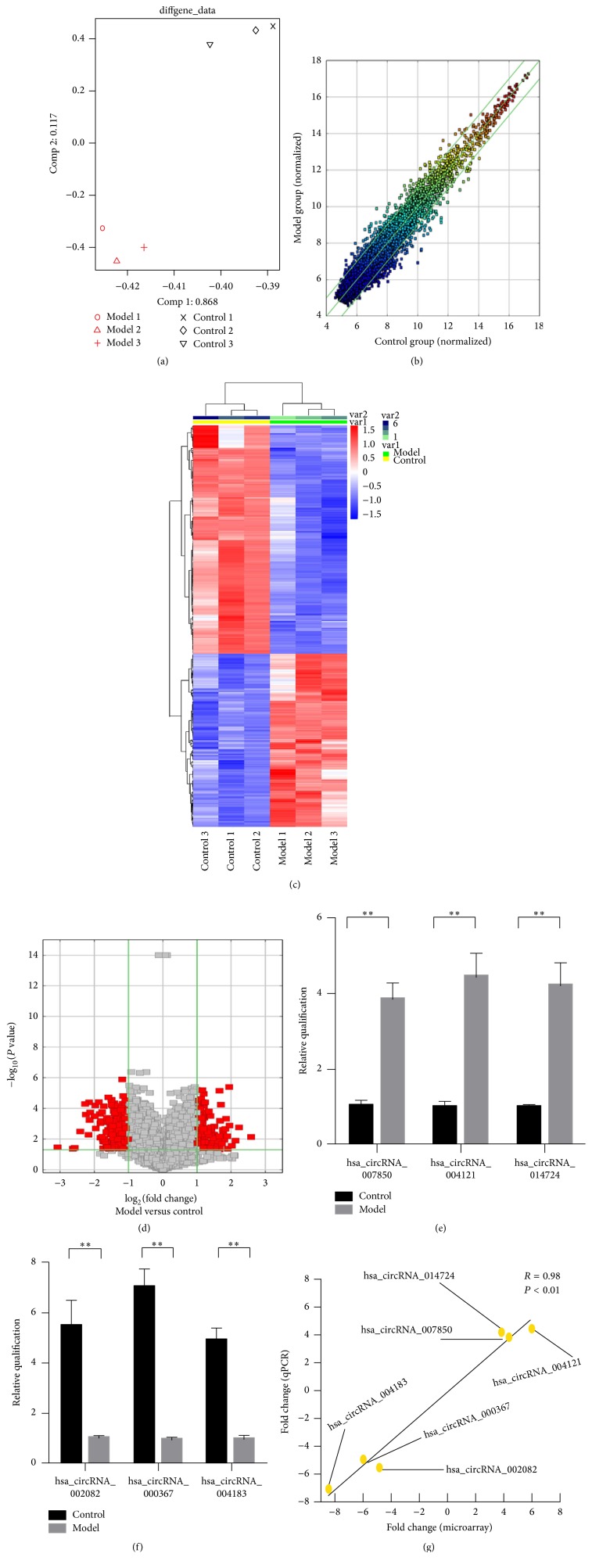
circRNA profiles differentiate the normal group from model group. (a) Principal component analysis (PCA) shows the difference between normal and model groups. (b) Scatter plots assess the variation of circRNA expression between two groups. The values plotted on *x* and *y* axes are the normalized signal values of each group (log2 scaled). Dots above the top green line and below the bottom green line represent differentially expressed circRNAs. (c, d) Unsupervised hierarchical clustering (c) and volcano plot (d) demonstrate the differential expression of circRNAs during hepatic steatosis. Both downregulated (green) and upregulated (red) circRNAs were visualized in the cluster. In similar, the red points in volcano plot represent the differentially expressed circRNAs with statistical significance. (e, f) Validation of the upregulated (e) and downregulated circRNAs (f) using QPCR. (g) The results of QPCR exhibit well consistence with those of microarray. Pairwise scatter plots reflect the fold changes (log2 transformed) of both microarray (horizontal axis) and the QPCR (vertical axis). The R stands for linear correlation coefficient. The presented results are expressed as means ± SD. ^*∗∗*^*P* < 0.01.

Using the Human circRNA Arrays platform with 13,617 labeled probes, we assessed the expression profiles of circRNA in normal and model groups (Figures 2(a) and 2(b)). A total of 357 circRNAs was determined to be dysregulated (Table 2), and distinctly separated samples into biologically interpretable groups on a basis of hierarchical clustering and volcano plot. Among these differentially expressed circRNAs, 154 circRNAs were identified to be upregulated more than 2-fold in steatotic HepG2 cells as compared to that in normal ones (Table 3) (Figures 2(c) and 2(d)). In contrast, 203 circRNAs exhibited the decreased level less than threshold (0.5-fold) during the progression of hepatic steatosis (Table 3) (Figures 2(c) and 2(d)).

Six circRNAs among these filtered ones were validated to be significantly different between the normal and model groups. In well consistence to the results of microarray hybridization, expression levels of hsa_circRNA_002082, hsa_circRNA_000367, and hsa_circRNA_004183 were downregulated in the model group rather than the normal one (*P* < 0.01, Figures 2(f) and 2(g)), while hsa_circRNA_007850, hsa_circRNA_004121, and hsa_circRNA_014724 showed an opposite expression pattern (*P* < 0.01, Figures 2(e) and 2(g)).

### 3.2. Transcriptional Regulation Characterized the Effects of circRNAs

**Figure 3:**
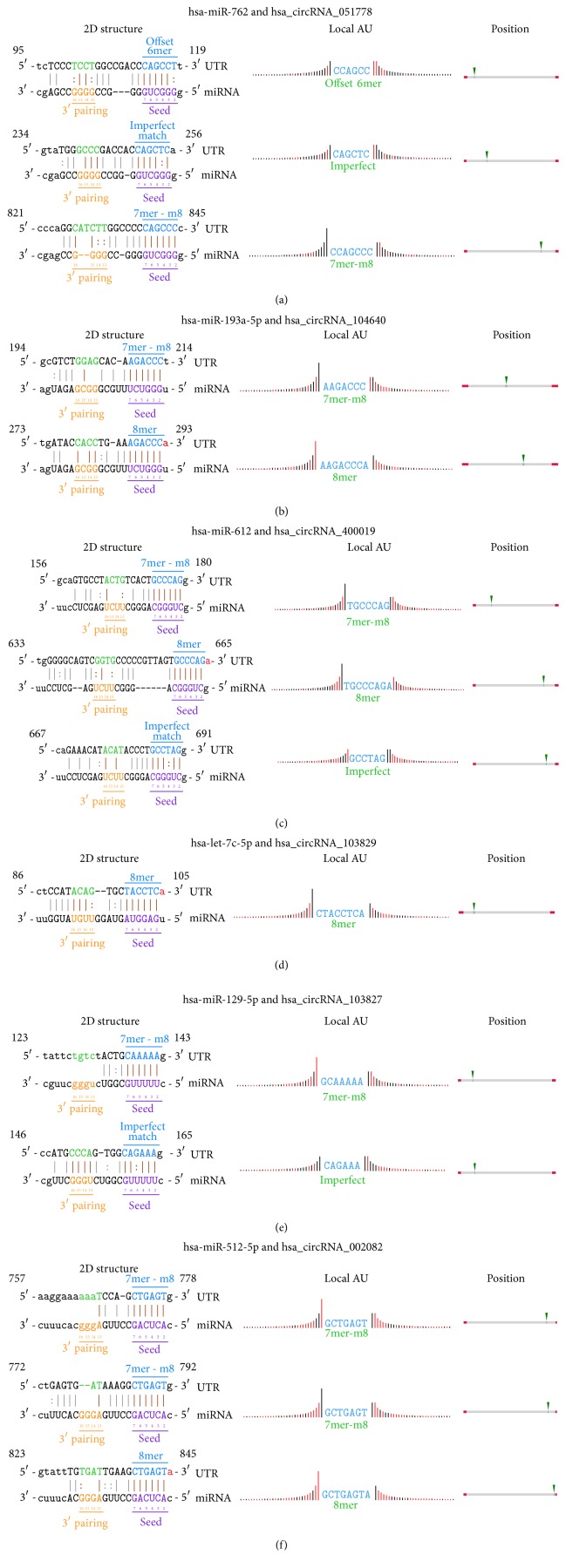
Target prediction of steatosis-related circRNAs. (a–f) Complementary binding of the top-six differentially expressed circRNAs and target miRNAs. The 2D structures show the complementation between circRNAs and miRNAs at both seed and 3′ pairing sequences (nucleotides 13–16). AU content 30 nt upstream and downstream of the seed sequence, respectively, is shown in the column of Local AU. Black bars reflect G/C and low accessibility, whereas red bars reflect A/U and high accessibility of seed sequence. Height of these bars shows the degree of accessibility. The column of position shows the location of microRNA response elements (MREs) in circRNAs.

According to the complimentary binding of microRNAs and MRE-containing sequences (Figure 3), 1,766 miRNAs and 17,876 mRNAs were predicted to be regulated by the differentially expressed circRNAs (Table 3). GO and pathway analyses further categorized these mRNAs depending on biological process and molecular function.

**Figure 4:**
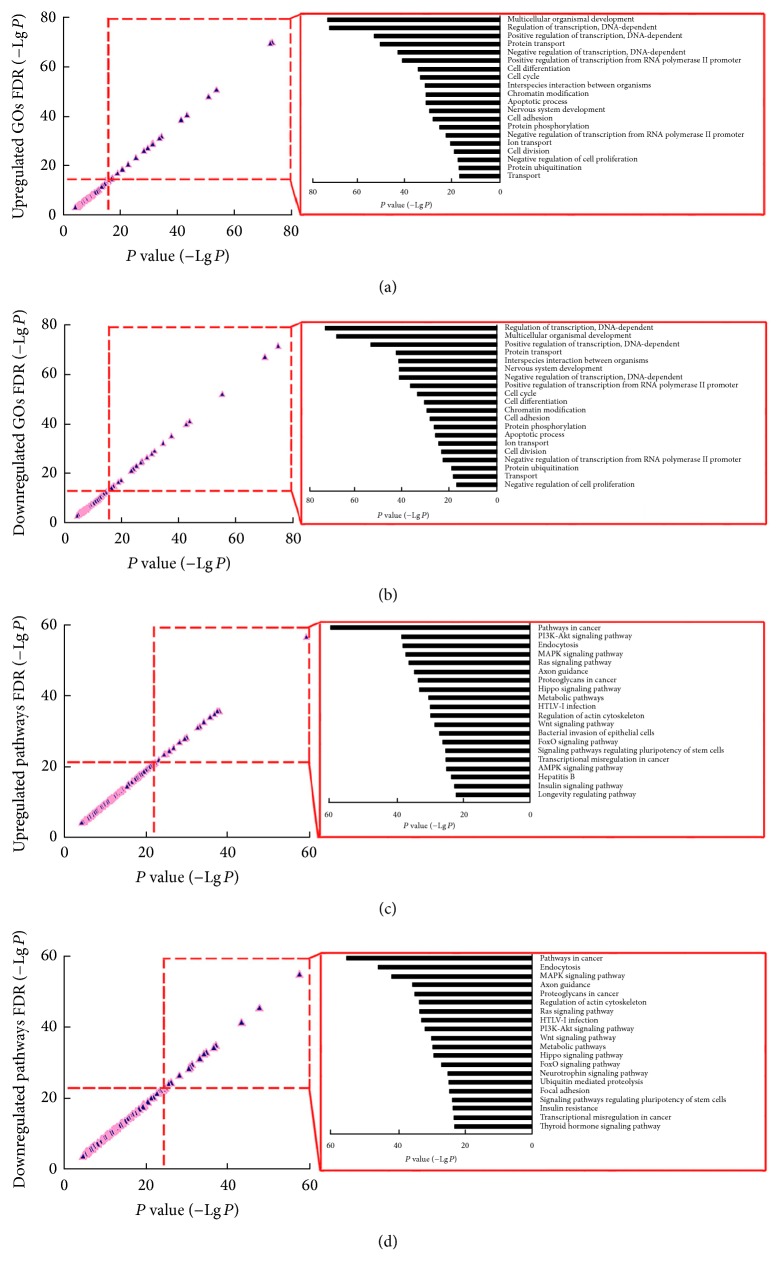
Functional analysis reveals the effect of steatosis-related circRNAs. (a, b) Upregulated (a) and downregulated gene ontologies (GOs) (b) show the effects of differentially expressed circRNAs with specific actions in transcriptional regulation. (c, d) Upregulated (c) and downregulated pathways (d) exhibit the regulatory role of differentially expressed circRNAs in signaling transduction.

After the filtration with thresholds of *P* value and FDR, 225 upregulated and 231 downregulated GOs revealed the functional effects of differentially expressed circRNAs. The top-ranking, downregulated GOs were regulation of transcription, DNA-dependent (GO: 0006355), multicellular organismal development (GO: 0007275), positive regulation of transcription, DNA-dependent (GO: 0045893), negative regulation of transcription, DNA-dependent (GO: 0045892), and positive regulation of transcription from RNA polymerase II promoter (GO: 0045944), and so forth (Figure 4(b)). On the other side, significantly upregulated GOs corresponding to circRNAs appeared to be multicellular organismal development (GO: 0007275), regulation of transcription, DNA-dependent (GO: 0006355), positive regulation of transcription, DNA-dependent (GO: 0045893), protein transport (GO: 0015031), negative regulation of transcription, DNA-dependent (GO: 0045892), and positive regulation of transcription from RNA polymerase II promoter (GO: 0045944), and so forth (Figure 4(a)). Transcriptional regulation, therefore, dominated both up- and downregulated GOs, suggesting a transcription-specific effect of steatosis-related circRNAs.

Pathway annotation provided us with another insight into the actions of circRNAs. As evaluated by the statistical significance and FDR, there were multiple up- and downregulated pathways with metabolic characteristics, including metabolic pathways (hsa01100), insulin signaling pathway (hsa04910), insulin resistance (hsa04931), and so forth (Figures 4(c) and 4(d)). Because of the HepG2 cells employed in our experiments, a lot of top-ranking pathways (i.e., pathways in cancer (hsa05200), proteoglycans in cancer (hsa05205), and transcriptional misregulation in cancer (hsa05202)) play essential roles in carcinogenesis, no matter in the up- or downregulated pathway sets (Figures 4(c) and 4(d)). Thus metabolic regulation was also indicated to underlie the effects of circRNAs.

Gene sets of GOs and pathways were then intersected for data mining. Interestingly, lipin 1 (LPIN1) was recognized to be the member of both downregulated transcription-regulating GO (regulation of transcription, DNA-dependent) and downregulated metabolic pathways. The bridging of GO and pathway qualified LPIN1 a critical part in circRNAs' regulation of hepatic steatosis.

### 3.3. circRNA_021412-miR-1972-LPIN1 Signaling Reflected the Regulatory Mechanisms Underlying Steatosis-Related circRNAs

**Figure 5:**
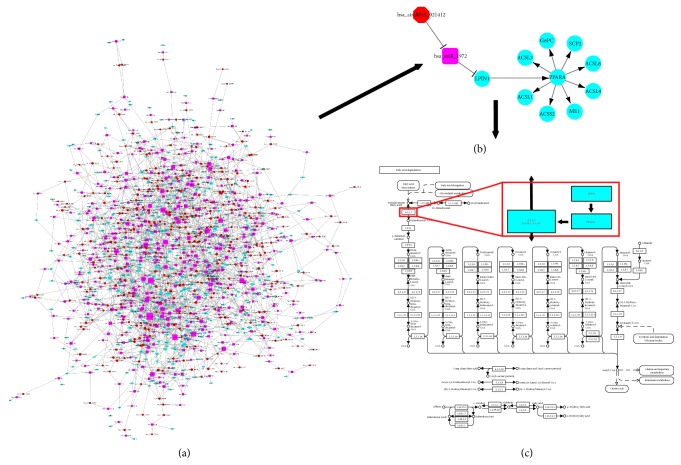
circRNA-miRNA-mRNA regulatory network uncovers the circRNA_021412-miR-1972-LPIN1 signaling underlying circRNAs' effects. (a) The circRNA-miRNA-mRNA network related to transcriptional regulation. Red cycles, violet squares, and blue cycles represent circRNAs, miRNAs, and mRNAs, respectively. The size of each symbol reflects its degree, which is scored by the number of downstream targets. (b) circRNA_021412-miR-1972-LPIN1 signaling within the circRNA-miRNA-mRNA network is recognized to underlie the actions of circRNAs. (c) The circRNA_021412-miR-1972-LPIN1 signaling controls the pathway of lipid degradation via PPAR*α*-induced ACSLs expression.

In order to outline the global action of circRNAs in transcriptional regulation, we selected the differentially expressed circRNAs, target miRNAs with high-complementary activity (≤5 miRNAs for each circRNA), and gene sets of transcription-regulating GOs for bioinformatic modeling. Afterwards, delineation of the circRNA-miRNA and miRNA-mRNA interactions constructed a circRNA-miRNA-mRNA regulatory network comprising 357 circRNAs, 1,766 miRNAs, and 17,876 mRNAs (Figure 5(a)). Characteristically, this multilayer, complex network was featured by signaling cascade, to a large extent, in multi-to-multi-manner.

**Figure 6:**
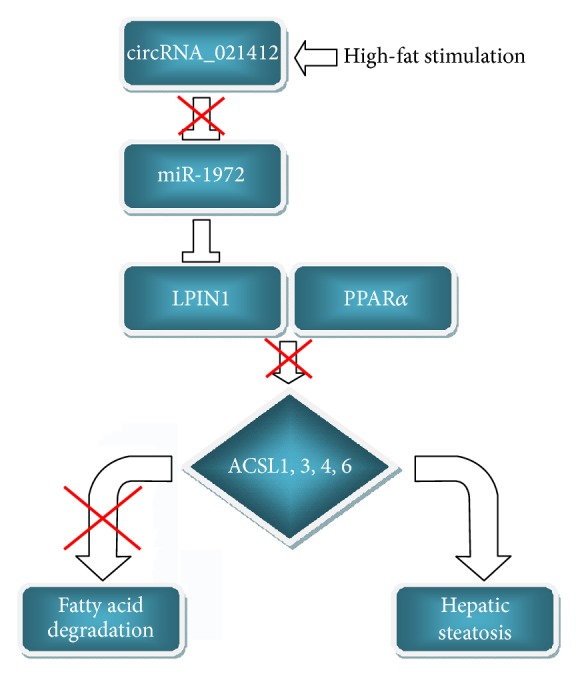
Diagram of circRNA_021412-based regulation of hepatic steatosis. Arrow, blunt-headed line, and red cross represent effects of activation, inhibition, and blocking, respectively.

Taking advantage of the circRNA-miRNA-mRNA network, LPIN1 was found to be successively regulated by hsa-circRNA_021412 and hsa-miR-1972. LPIN1 also serves as the coactivator of transcription factors (PPAR*α*, PGC-1*α*), both of which take the central place during hepatic steatosis. Thus signaling cascade of circRNA_021412/miR-1972/LPIN1 induced the expression of 8 steatosis-related genes, including* ACSL1*,* ACSL3*,* ACSL4*,* ACSL6*,* ACSS2*,* G6PC*,* ME1*, and* SCP2* (Figure 5(b)), via PPAR*α* activation [36]. As defined by KEGG database, ACSLs among these ones initiate the process of lipid degradation (Figure 5(c)). Resultantly, the circRNA_021412/miR-1972/LPIN1 signaling uncovered an important mechanism that underlay the transcription-dependent role of circRNA in metabolic regulation and steatosis induction (Figure 6).

## 4. Discussion

Mimicking high-fat diet that induces NAFLD, HepG2 cells were administrated using the mixture of saturated (palmitate) and unsaturated fatty acids (oleate) at a ratio of 1 : 2 [37]. Both the lipid droplets positive to Oil Red-O staining and the significant increase in TG content indicated the establishment of hepatic steatosis. When evaluated, by the microarray profiling, 154 up- and 203 downregulated circRNAs, representing 2.6% of the total amount, and characterized the model group as compared to those of the normal group. Moreover, two groups of HepG2 cells, with or without steatosis, were distinctly differentiated by these circRNAs on the basis of PCA, unsupervised hierarchical clustering, and volcano plot, respectively. circRNAs, therefore, are suggested to play a pathological role underlying the hepatic steatogenesis.

circRNAs have been widely accepted to function as the sponge for miRNAs and the competing endogenous RNAs for mRNAs, mainly on the basis of complementation between MRE and “seed sequence” [12]. Thus joint analysis of large-scale profiling coupled with computational prediction represents a powerful approach to elucidate the possible biological roles of circRNAs. Accordingly, differentially expressed circRNAs were subjected to target scanning by circRNA-miRNA, and miRNA-mRNA interaction. A total of 17,876 target mRNAs were then mapped to databases of DAVID and KEGG, respectively, for GO and pathway analyses. In result, GOs related to gene transcription, such as regulation of transcription, DNA-dependent (GO: 0006355), positive regulation of transcription, DNA-dependent (GO: 0045893), positive regulation of transcription from RNA polymerase II promoter (GO: 0045944), negative regulation of transcription, DNA-dependent (GO: 0045892), and negative regulation of transcription from RNA polymerase II promoter (GO: 0000122), dominated the set of upregulated GOs. Similar phenomenon could be observed in the set of downregulated GOs. Dramatically, sets of up- and downregulated GOs shared most of these top-ranking GOs. In consistence to their regulatory actions in metabolism [16], development [13], and carcinogenesis [38], circRNAs are finally highlighted to be robust transcriptional regulators during steatogenesis, probably in a counteracting manner. Abnormal expression of circRNAs may disrupt the balance in transcription and lead to the TG deposition (steatosis). Except for the signaling pathways related to its malignant origination (pathways in cancer, pathways in cancer, transcriptional misregulation in cancer, etc.), steatotic HepG2 cells were featured by dysregulation of metabolic pathways (hsa01100), insulin signaling pathway (hsa04910), and insulin resistance (hsa04931). Taken together, circRNAs are proposed to control metabolic pathways, and related ones, in hepatic steatosis by modulating the transcription of critical genes.

For the purpose of taking deep insight into circRNAs' action model, differentially expressed circRNAs, target miRNAs, and downstream genes within the transcription-regulating GOs (up- and downregulated GOs of regulation of transcription, DNA-dependent) were integrated to construct a circRNA-miRNA-mRNA network. Briefly, the circRNA-miRNA-mRNA network was composed of 357 circRNAs, 1,766 miRNAs, and 17,876 mRNAs, indicating the steatogenic mechanisms with characteristics of signaling cascaded, multi-to-multi-regulation. LPIN1 among this network was identified to be the only member in both downregulated, transcription-regulating GO and downregulated metabolic pathways. Therefore, LPIN1 may serve as the critical agent that mediates the transcriptional regulatory effect of circRNAs on metabolic pathways. Has_circRNA_021412 and miR-1972 with LPIN1-regulating activity, and LPIN1 itself, were recognized to take the central place in circRNA-miRNA-mRNA network.

Interestingly, interaction of these circRNA, miRNA, and LPIN1 reveals some novel, yet important, mechanisms during hepatic steatosis. Being assessed by array hybridization and QPCR, has_circRNA_021412 experienced significant downregulation after high-fat stimulation. The decreased expression of circRNA attenuates its competitive inhibition to miRNA. As a result, reactivated miR-1972 induces the transcriptional and/or translational reduction in LPIN1 level. LPIN1 has been confirmed to play a dual function as phosphatidic acid phosphohydrolase (PAP) and transcriptional regulator, respectively, in hepatocytes [39, 40]. First, it suppresses the lipogenic program by catalyzing the penultimate step of TG synthesis in endoplasmic reticulum [34]. Second, direct interaction between LPIN1 and transcription factors (PPAR*α*, PGC-1*α*) amplifies the hepatic PGC-1*α*/PPAR*α* signaling in steatosis-related gene expression [40]. Indeed, 8 steatosis-related genes [41] of the metabolic pathways were recognized to be transcriptionally induced by LPIN1 in our experiments. Members of long chain acyl-CoA synthetase (ACSL) family (ACSL1, ACSL3, ACSL4, and ACSL6) dramatically prevailed in these genes. Because of ACSLs' essential role in fatty acid oxidation [42, 43], LPIN1 is capable of selectively activating fatty acid oxidation and mitochondrial oxidative phosphorylation, which have already been proved by gain-of-function and loss-of-function strategies [40, 44–46]. Downregulated LPIN1, however, prevent the fatty acid degradation in an ACSL-dependent way. Furthermore, LPIN1 is verified to act as the key component of multiple signaling transductions that are deeply involved in lipid homeostasis, including SIRT1/AMPK signaling [47], mammalian target of rapamycin complex 1 (mTORC1)/SREBP signaling [48], NF-E2-related factor 1 (Nrf1) signaling [49], and hepatocyte nuclear factor 4 *α* (HNF4*α*) signaling [50]. Because of these reasons, abnormality in circRNA_021412/miR-1972/LPIN1 signaling cascade contributes to the hepatic steatosis via disrupting the balance of lipogenesis and catalytic separation.

In conclusion, genome-scale dysregulation of circRNAs is associated with hepatic steatosis. Transcriptional regulation characterizes their actions and is suggested to mediate the modulation of metabolic pathways. circRNA_021412/miR-1972/LPIN1 signaling may reflect the critical mechanism underlying circRNA-related fatty acid dysmetabolism, which results in the cellular steatogenesis.

## Figures and Tables

**Table 1:** Primers for qPCR.

Gene	Primer sequence (5′-3′)		Product length (nt)
hsa_circ_002082	F: CCAGCTGAGTGATAAAGGCTGA	R: TCGTTCTTCCGCTCAAATCCT	158
hsa_circ_004183	F: CGTCCATTCCACGAGGTTCT	R: CCTCTGACGCAGGGTTTCC	113
hsa_circ_000367	F: CAATTGCACATTCCCTGCACT	R: CGTATGGAATGGACCTGGACA	114
hsa_circ_007850	F: CAGAAAAGAAGGGGAAATTACAG	R: TGTATGTGCAGGATCTCTGGG	106
hsa_circ_004121	F: CTTTGCTGGGAACATCAACAGA	R: ACTGGGTCTGTCTTCATCGG	112
hsa_circ_014724	F: TCGAAATTAGCCGGACCCAG	R: GATGATATCTGCAATAGTCTTGGC	144
GAPDH	F: TCAAGGCTGAGAACGGGAAG	R: TGGACTCCACGACGTACTCA	117

**Table 2:** Predicted targets of top-20 differentially expressed circRNAs.

Gene	Regulation	Location	Target				
hsa_circRNA_400019	Up	chr11	hsa-miR-1298-3p	hsa-miR-612	hsa-miR-204-3p	hsa-miR-20b-3p	hsa-miR-135a-3p
hsa_circRNA_006560	Up	chr3	hsa-miR-6751-5p	hsa-miR-3662	hsa-miR-4709-3p	hsa-miR-6831-5p	hsa-miR-98-5p
hsa_circRNA_103561	Up	chr3	hsa-miR-888-3p	hsa-miR-520c-3p	hsa-miR-520b	hsa-miR-373-3p	hsa-miR-372-3p
hsa_circRNA_014724	Up	chr1	hsa-miR-5009-5p	hsa-miR-8058	hsa-miR-378h	hsa-miR-2681-3p	hsa-miR-378c
hsa_circRNA_104640	Up	chr8	hsa-miR-193a-5p	hsa-miR-520g-3p	hsa-miR-520h	hsa-miR-508-5p	hsa-miR-519d-5p
hsa_circRNA_403044	Up	chr3	hsa-miR-3121-5p	hsa-miR-3692-3p	hsa-miR-6504-3p	hsa-miR-1324	hsa-miR-4436a
hsa_circRNA_004121	Up	chr2	hsa-miR-1270	hsa-miR-4742-5p	hsa-miR-4446-3p	hsa-miR-6830-5p	hsa-miR-4677-5p
hsa_circRNA_100983	Up	chr11	hsa-miR-376a-2-5p	hsa-miR-873-5p	hsa-miR-765	hsa-miR-576-3p	hsa-miR-423-3p
hsa_circRNA_007850	Up	chr9	hsa-miR-6758-5p	hsa-miR-6856-5p	hsa-miR-4668-5p	hsa-miR-4773	hsa-miR-3976
sa_circRNA_051778	Up	chr19	hsa-miR-6762-5p	hsa-miR-762	hsa-miR-4697-5p	hsa-miR-4739	hsa-miR-4640-5p
hsa_circRNA_000317	Down	chr11	hsa-miR-659-5p	hsa-miR-3649	hsa-miR-365a-5p	hsa-miR-6509-5p	hsa-miR-6073
hsa_circRNA_103117	Down	chr21	hsa-miR-153-5p	hsa-miR-152-5p	hsa-miR-26b-3p	hsa-miR-513a-3p	hsa-miR-424-5p
hsa_circRNA_103829	Down	chr5	hsa-miR-625-3p	hsa-miR-129-5p	hsa-miR-532-5p	hsa-miR-548c-3p	hsa-let-7c-5p
hsa_circRNA_001729	Down	chr16	hsa-miR-367-3p	hsa-miR-363-3p	hsa-miR-323a-3p		
hsa_circRNA_000367	Down	chr11	hsa-miR-331-3p	hsa-miR-4646-5p	hsa-miR-4797-5p	hsa-miR-3919	hsa-miR-3190-3p
hsa_circRNA_082680	Down	chr7	hsa-miR-4518	hsa-miR-6851-5p	hsa-miR-6863	hsa-miR-373-3p	hsa-miR-4695-3p
hsa_circRNA_004183	Down	chr10	hsa-miR-7162-5p	hsa-miR-6875-3p	hsa-miR-516b-3p	hsa-miR-516a-3p	hsa-miR-4687-3p
hsa_circRNA_101837	Down	chr16	hsa-miR-766-5p	hsa-miR-509-5p	hsa-miR-18b-5p	hsa-miR-18a-5p	hsa-miR-380-3p
hsa_circRNA_103827	Down	chr5	hsa-miR-411-5p	hsa-miR-625-3p	hsa-miR-129-5p	hsa-miR-205-5p	hsa-miR-532-5p
hsa_circRNA_002082	Down	chr11	hsa-miR-512-5p	hsa-miR-4773	hsa-miR-3611	hsa-miR-4742-3p	hsa-miR-6887-3p

**Table 3:** Enumeration data of differentially expressed circRNAs and target miRNAs/mRNAs.

	circRNA	miRNA	mRNA
Up	154	768	8448
Down	203	1013	9428
